# Whole-Body [^18^F]FDG PET/CT Can Alter Diagnosis in Patients with Suspected Rheumatic Disease

**DOI:** 10.3390/diagnostics11112073

**Published:** 2021-11-09

**Authors:** Matthias Fröhlich, Sebastian Serfling, Takahiro Higuchi, Martin G. Pomper, Steven P. Rowe, Marc Schmalzing, Hans-Peter Tony, Michael Gernert, Patrick-Pascal Strunz, Jan Portegys, Eva-Christina Schwaneck, Ottar Gadeholt, Alexander Weich, Andreas K. Buck, Thorsten A. Bley, Konstanze V. Guggenberger, Rudolf A. Werner

**Affiliations:** 1 Department of Internal Medicine II, Rheumatology/Clinical Immunology, University Hospital Würzburg, 97080 Würzburg, Germany; schmalzing_m@ukw.de (M.S.); tony_h@ukw.de (H.-P.T.); gernert_m1@ukw.de (M.G.); strunz_p@ukw.de (P.-P.S.); portegys_j@ukw.de (J.P.); 2 Comprehensive Heart Failure Center, Department of Nuclear Medicine, University Hospital Würzburg, 97080 Würzburg, Germany; serfling_s1@ukw.de (S.S.); thiguchi@me.com (T.H.); buck_a@ukw.de (A.K.B.); werner_r1@ukw.de (R.A.W.); 3 Graduate School of Medicine, Dentistry and Pharmaceutical Sciences, Okayama University, Okayama 700-8530, Japan; 4 The Russell H Morgan Department of Radiology and Radiological Science, Division of Nuclear Medicine, Baltimore, MD 21287, USA; mpomper@jhmi.edu (M.G.P.); srowe8@jhmi.edu (S.P.R.); 5 Asklepios Klinik Altona, Rheumatology and Clinical Immunology, 22763 Hamburg, Germany; e.schwaneck@asklepios.com; 6 Rheumatologische Schwerpunkt Praxis Würzburg, 97070 Würzburg, Germany; ottar.gadeholt@gmail.com; 7 Gastroenterology, Department of Internal Medicine II, University Hospital Würzburg, 79080 Würzburg, Germany; weich_a@ukw.de; 8 Department of Diagnostic and Interventional Radiology, University Hospital Würzburg, 97080 Würzburg, Germany; bley_t@ukw.de (T.A.B.); guggenberg_k@ukw.de (K.V.G.)

**Keywords:** giant cell arteritis, GCA, [^18^F]FDG PET/CT, vasculature, inflammation, polymyalgia rheumatica, PMR, vasculitis

## Abstract

The 2-deoxy-d-[^18^F]fluoro-D-glucose (FDG) positron emission tomography/computed tomography (PET/CT) is widely utilized to assess the vascular and articular inflammatory burden of patients with a suspected diagnosis of rheumatic disease. We aimed to elucidate the impact of [^18^F]FDG PET/CT on change in initially suspected diagnosis in patients at the time of the scan. Thirty-four patients, who had undergone [^18^F]FDG PET/CT, were enrolled and the initially suspected diagnosis prior to [^18^F]FDG PET/CT was compared to the final diagnosis. In addition, a semi-quantitative analysis including vessel wall-to-liver (VLR) and joint-to-liver (JLR) ratios was also conducted. Prior to [^18^F]FDG PET/CT, 22/34 (64.7%) of patients did not have an established diagnosis, whereas in 7/34 (20.6%), polymyalgia rheumatica (PMR) was suspected, and in 5/34 (14.7%), giant cell arteritis (GCA) was suspected by the referring rheumatologists. After [^18^F]FDG PET/CT, the diagnosis was GCA in 19/34 (55.9%), combined GCA and PMR (GCA + PMR) in 9/34 (26.5%) and PMR in the remaining 6/34 (17.6%). As such, [^18^F]FDG PET/CT altered suspected diagnosis in 28/34 (82.4%), including in all unclear cases. VLR of patients whose final diagnosis was GCA tended to be significantly higher when compared to VLR in PMR (GCA, 1.01 ± 0.08 (95%CI, 0.95–1.1) vs. PMR, 0.92 ± 0.1 (95%CI, 0.85–0.99), *p* = 0.07), but not when compared to PMR + GCA (1.04 ± 0.14 (95%CI, 0.95–1.13), *p* = 1). JLR of individuals finally diagnosed with PMR (0.94 ± 0.16, (95%CI, 0.83–1.06)), however, was significantly increased relative to JLR in GCA (0.58 ± 0.04 (95%CI, 0.55–0.61)) and GCA + PMR (0.64 ± 0.09 (95%CI, 0.57–0.71); *p* < 0.0001, respectively). In individuals with a suspected diagnosis of rheumatic disease, an inflammatory-directed [^18^F]FDG PET/CT can alter diagnosis in the majority of the cases, particularly in subjects who were referred because of diagnostic uncertainty. Semi-quantitative assessment may be helpful in establishing a final diagnosis of PMR, supporting the notion that a quantitative whole-body read-out may be useful in unclear cases.

## 1. Introduction

Giant cell arteritis (GCA) is the most common form of systemic vasculitis in adults. It is characterized by a heterogeneous picture of various symptoms, including headache, visual disturbances, vision loss, claudication of the jaw or even more nonspecific symptoms such as fever, weight loss or fatigue [1,2,3]. In addition, some patients show symptoms of polymyalgia rheumatica (PMR) [4,5], with a mostly symmetrical inflammation of the extracapsular structures, primarily of the shoulders and pelvic girdle [6]. To date, no specific clinical or laboratory parameter exists to fully distinguish between GCA and PMR [7]. However, non-invasive, whole-body positron emission tomography/computed tomography (PET/CT) simultaneously assessing inflammatory burden in the vessels and joints may be particularly useful for this purpose [8]. As such, the PET glucose consumption biomarker 2-deoxy-d-[^18^F]fluoro-D-glucose ([^18^F]FDG) was used in patients with a suspected diagnosis of GCA and/or PMR [9,10]. We aimed to investigate whether visual and semi-quantitative analyses of [^18^F]FDG PET/CT in patients with a suspected diagnosis of rheumatic disease could substantially alter initial diagnosis, thereby increasing diagnostic confidence of the referring rheumatologist [11].

## 2. Materials and Methods

### 2.1. Patients

Thirty-four patients of the Department of Internal Medicine II, Section Rheumatology, University Hospital Würzburg, who had undergone [^18^F]FDG PET/CT to establish a diagnosis, were analyzed. C-reactive protein (CRP) and white blood cell count (WBC) at the time of scan were also collected. This retrospective analysis included patients >50 years of age who were referred to us as a tertiary center for rheumatology for clarification of an unclear inflammatory constellation after primary diagnostic workup had excluded malignant or infectious diseases, and rheumatologic workup had found no evidence of rheumatoid arthritis, spondylarthritis, anti-neutrophil cytoplasmatic antibody-associated (ANCA) vasculitis or connective tissue disease. In addition, patients with suspected GCA and/or PMR after the failure of standard diagnostic approaches (ultrasound and magnetic resonance imaging (MRI)) or because of suspected PMR or GCA with atypical symptoms or atypical disease course were examined. For cranial symptoms, the 1990 American College of Rheumatology (ACR) criteria were used to classify as GCA [12]. The diagnosis of PMR was based on meeting the 2012 EULAR provisional classification criteria for PMR [7]. Subjects were grouped as either having an unclear diagnosis, GCA, PMR or both (GCA + PMR). [^18^F]FDG PET/CTs of 10 randomly selected patients afflicted with an oncological disease were used as a control group. None of these controls had concomitant known GCA and/or PMR or signs of inflammation (defined as CRP within the normal reference range). To assess change in diagnosis, [^18^F]FDG PET/CT-based findings were compared to the initial diagnosis established by the board-certified referring rheumatologist. The results of [^18^F]FDG PET/CT were also confirmed in a 3-months follow-up visit by a board-certified rheumatologist.

### 2.2. PET/CT Acquisition

[^18^F]FDG was synthesized in-house with a 16 MeV Cyclotron (Würzburg; GE PET trace 6; GE Healthcare, Milwaukee, WI, USA). Scans were performed on a PET/CT scanner (Siemens Biograph mCT 64 or mCT 128, Siemens, Knoxville, TN, USA). Patients fasted at least 6 h prior to injection of 289.1 ± 38.5 MBq [^18^F]FDG. PET/CT scans were acquired after 60 min post-injection, using non-contrast-enhanced CT with CARE Dose 4D with the following parameters: 160 mAs, 120 kV, 512 × 512 matrix, 5 mm slice thickness, slice collimation 64 × 0.6 mm [13]. PET data were reconstructed according to standard protocols as described in [13]. Quality controls of PET and CT were conducted on a regular basis.

### 2.3. PET/CT Analysis

PET images were analyzed by three physicians experienced in reading PET/CT using a dedicated workstation (Syngo.Via; V50B; Siemens Healthcare, Erlangen, Germany), which allowed simultaneous and fused review of PET and CT data. PET, CT and hybrid PET/CT image overlays were assessed in all patients. A semi-quantitative read-out was performed, as described in [14]. In order to assess inflammatory activity in vessels and joints, a total of 782 volumes of interest (VOIs) were drawn to provide mean standardized uptake values (SUV_mean_). For vessel analysis, circular volumes of interest (VOIs) were manually defined for ascending aorta, aortic arch, descending and abdominal aorta, innominate artery (brachiocephalic trunk), both carotid arteries, both subclavian arteries and iliac arteries [8]. For joints, circular VOIs were placed in the following regions: shoulders, acromioclavicular (AC) joints, sternoclavicular (SC) joints, greater trochanters, ischial tuberosities and (averaged uptake) of interspinal ligaments of lumbar vertebrae 3–5 [8]. Moreover, VOIs were placed on healthy liver tissue [14], serving as reference according to current guidelines [8]. Vessel wall-to-liver (VLR) and joint-to-liver ratio (JLR) were then calculated by dividing the vessel or joint uptake by the liver. In addition, VOIs were also placed in the blood pool, with jugular veins serving as background tissue [8]. VOIs were defined by one of two readers (RS, KG) and then verified by a second reader (RAW). Similar procedures providing JLR and VLR were performed in the control group.

### 2.4. Statistical Analysis

For statistical analysis, Prism (version 8.4.2 (GraphPad, San Diego, CA, USA)) was used. For continuous variables, mean ± standard deviations are presented. For comparison of VLR and JLR, the Mann–Whitney U-test was used between the different groups (GCA, PMR, GCA + PMR, controls). Bonferroni adjustment was also performed. A *p*-value of <0.05 was considered to be statistically significant [14].

## 3. Results

### 3.1. [^18^F]FDG PET/CT Can Alter Diagnosis in Individuals with Suspected Diagnosis of Rheumatic Disease

Patients presented with various symptoms, e.g., weakness in 15/34 (44.1%), arthritis/arthralgia in 13/34 (38.2%), weight loss in 12/34 (35.3%), night sweats in 11/34 (32.2%), or fever in 10/34 (29.4%). A total of 30/34 (88.2%) patients had no therapy at the time of [^18^F]FDG PET/CT, whereas, in the remaining four (11.8%), low-dose prednisolone had already been initiated by the referring primary care physician due to unclear joint symptoms. The patients’ characteristics are summarized in Table 1.

**Figure 1:**
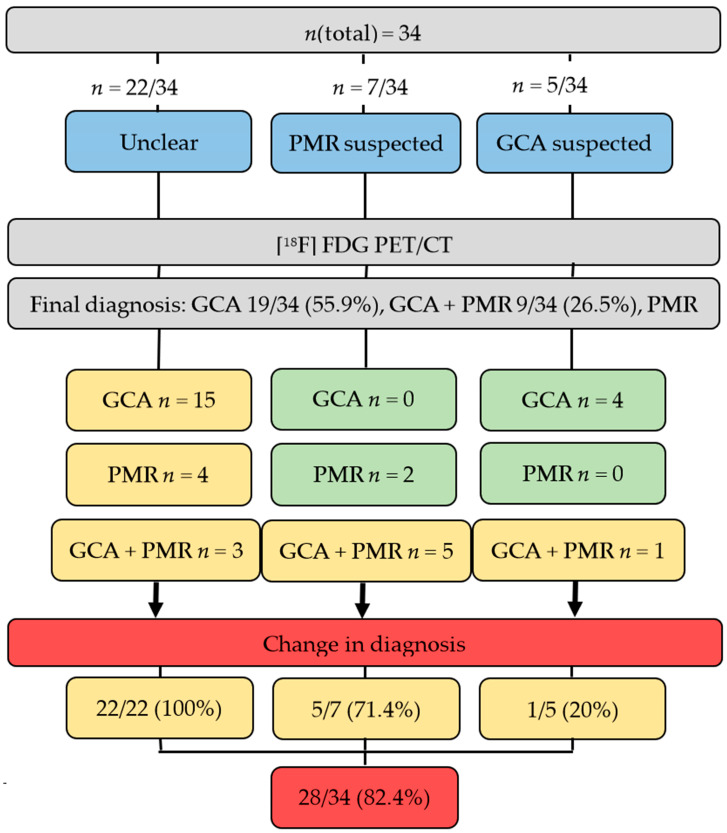
Thirty-four patients were examined by [^18^F]fluorodeoxyglucose (FDG) positron emission tomography/computed tomography (PET/CT) with unclear diagnosis or suspected giant cell arteritis (GCA) or polymyalgia rheumatica (PMR). In 28/34 (82.4%), [^18^F]FDG PET/CT was able to establish the diagnosis (red). In the remaining 6/34 (17.6%), [^18^F]FDG PET/CT had no additional benefit (green). The greatest benefit was recorded in an unclear situation (22/22 [100%]), with the vast majority having a final diagnosis of GCA (15/22 [68.2%]), followed by PMR in 4/22 (18.2%) and GCA + PMR in the remaining 3/22 (13.6%) (yellow).

**Figure 2:**
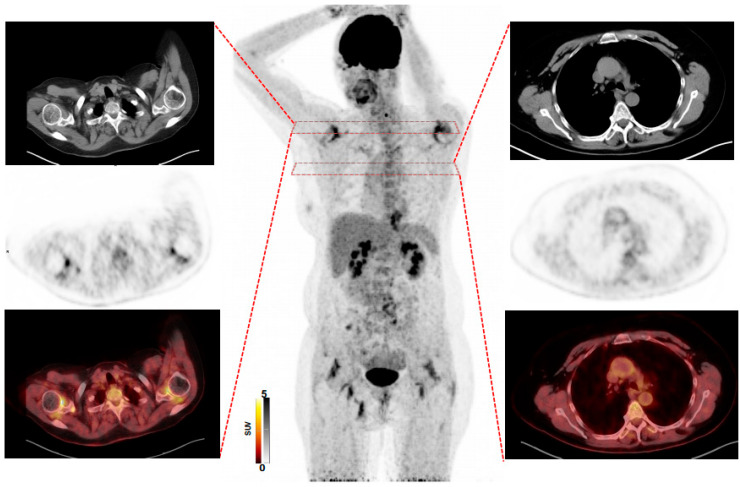
A 58-year-old woman with typical symptoms of polymyalgia rheumatica, including shoulder and hip girdle pain. Maximum intensity projection (**middle**) revealed intense uptake in multiple joints and in the thoracic aorta. Transaxial CT, PET and PET/CT revealed intense uptake in the AC joints (**left**) and aorta ascendens (**right**), suggestive for combined polymyalgia rheumatica and giant cell arteritis.

Prior to [^18^F]FDG PET/CT, 22/34 (64.7%) of patients had an unclear diagnosis, whereas in 7/34 (20.6%) PMR and in 5/34 (14.7%), GCA was suspected by rheumatologists. After the scan, the established diagnosis was GCA in 19/34 (55.9%), GCA + PMR in 9/34 (26.5%) and PMR alone in the remaining 6/34 (17.6%). As such, [^18^F]FDG PET/CT changed the suspected diagnosis in 28/34 (82.4%) of patients. In the remaining 6/34 (17.6%), however, the already clinically suspected diagnosis of GCA and/or PMR was confirmed. In 5/7 (71.4%) cases, in which PMR was suspected, GCA was detected additionally. However, if GCA alone was suspected, change in diagnosis was recorded only in 1/5 (20%). Of note, the scan established the diagnosis in all unclear cases (22/22 (100%)), with the vast majority having a final diagnosis of GCA (15/22 (68.2%)), followed by PMR, 4/22 (18.2%) and GCA + PMR (3/22 (13.6%)), supporting the notion that [^18^F]FDG PET/CT has the greatest benefit in subjects without disease-specific manifestations (Figure 1). Figure 2 shows an [^18^F]FDG PET/CT of a patient who had typical symptoms of PMR. The scan revealed findings suggestive for combined disease (GCA + PMR).

### 3.2. Semi-Quantitative Assessment Helps in Establishing Final Diagnosis, in Particular for PMR

**Figure 3:**
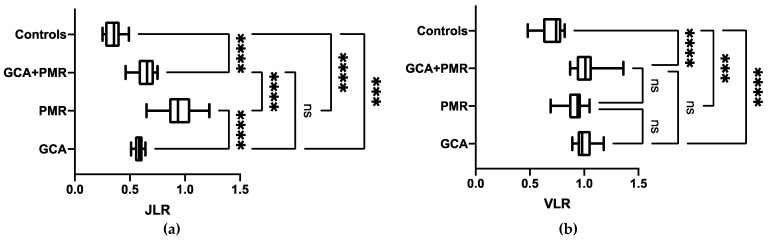
(**a**) The vessel wall-to-liver ratio (VLR) showed no significant differences between patients with final diagnosis of giant cell arteritis (GCA) + polymyalgia rheumatica (PMR) vs. PMR alone or GCA alone. Relative to the control group, significant differences were reached for all three subgroups. (**b**) The joint-to-liver ratio (JLR), however, was significantly increased in patients with PMR relative to GCA alone or GCA + PMR. Significant differences were also reached for all three subgroups when compared to controls. *** *p* = 0.0001; **** *p* < 0.0001; ns = not significant.

Table 2 provides an overview of semi-quantitative parameters. VLR of patients finally diagnosed with GCA tended to be significantly higher when compared to VLR in PMR (GCA, 1.01 ± 0.08 (95%CI, 0.95–1.1) vs. PMR, 0.92 ± 0.1 (95%CI, 0.85–0.99), *p* = 0.07), but not when compared to PMR + GCA (1.04 ± 0.14 (95%CI, 0.95–1.13), *p* = 1). JLR of individuals finally diagnosed with PMR (0.94 ± 0.16, (95%CI, 0.83–1.06)), however, was significantly increased relative to JLR in GCA (0.58 ± 0.04 (95%CI, 0.55–0.61)) and GCA + PMR (0.64 ± 0.09 (95%CI, 0.57–0.71), *p* < 0.0001, respectively). VLR of controls was 0.70 ± 0.12 (95%CI, 0.62–0.77) and JLR was 0.35 ± 0.08 (95%CI, 0.29–0.41), which were significantly lower when compared to GCA, PMR, or GCA + PMR (*p* ≤ 0.0001, respectively). The results are shown in Figure 3.

As such, a semi-quantitative analysis of the joints may be helpful to distinguish between patients with PMR vs. GCA or combined disease. Similar results were obtained when the blood pool was used as reference tissue. Derived Joint to Blood Pool Ratios of individuals finally diagnosed with PMR (1.35 ± 0.23, (95%CI, 1.18–1.51)) were again significantly increased relative to blood pool-based ratios in GCA alone (0.88 ± 0.07, (95%CI, 0.83–0.93)) or GCA + PMR (0.96 ± 0.14, (95%CI, 0.85–1.06), *p* < 0.001, respectively; Supplementary Materials Figure S1b).

## 4. Discussion

This study investigated the value of [^18^F]FDG PET to establish the diagnosis in 34 patients over 50 years of age with suspected rheumatic diseases in the difficult clinical situation where malignancy, infectious cause and serious rheumatologic diseases (i.e., rheumatoid arthritis, spondylarthritis, ANCA-associated vasculitis, or connective tissue disease) had already been ruled out or in whom GCA and/or PMR were suspected but the diagnosis could not be confirmed by standard procedures. In those patients, [^18^F]FDG PET changed the final diagnosis in >82% of the cases. Of note, in all of the 22 patients who were referred because of diagnostic uncertainty, a final diagnosis was established, supporting the notion that [^18^F]FDG PET/CT is a helpful tool even in such challenging scenarios. Moreover, in a semi-quantitative analysis placing 782 VOIs in the vessels and joints, the newly introduced parameter JLR was able to distinguish between patients with a final diagnosis of PMR and subjects with GCA or combined disease. Using VLR, however, only a trend for differentiating between GCA and PMR was found. As such, semi-quantitative assessment may be helpful in establishing a final diagnosis of PMR, supporting the notion that a quantitative whole-body read-out may be performed in unclear cases.

Prior studies already reported on an improved diagnostic certainty with [^18^F]FDG PET/CT in the context of an unclear diagnosis, a scenario referred to as inflammation of unknown origin (IUO) [10,15,16]. For instance, Schoenau et al. demonstrated that in IUO, [^18^F]FDG PET/CT was helpful in ascertaining the diagnosis in up to 71% of the patients, with GCA followed by PMR. Furthermore, in this study, age over 50, elevated C-reactive protein and absence of fever were identified as predictors of a diagnostic [^18^F]FDG PET/CT [10]. Such a constellation of symptoms, however, applies to a relatively large portion of patients afflicted with rheumatic disease [17]. As such, results of the present study reporting on an established diagnosis in 100% of unclear cases further confirm the usefulness of [^18^F]FDG PET/CT in such challenging scenarios.

Only 8/34 (23.5%) of the investigated patients had symptoms related to the skull, including visual loss, headache and jaw claudication. GCA, however, is often only considered in the presence of those symptoms, in particular as the American College of Rheumatology Criteria for GCA are not appropriate for classifying patients with large-vessel GCA (LV GCA), which also involves the aorta and its branches [12]. The 2018 EULAR recommendations for the management of large-vessel vasculitis also focus primarily on cranial symptoms in GCA but also mention that purely constitutional symptoms may also indicate GCA [18]. This may partially explain why only 5/34 (14.7%) in our cohort was suspected as GCA prior to [^18^F]FDG PET/CT. Another explanation could be that [^18^F]FDG PET/CT is still not a diagnostic tool of first choice for suspected rheumatic disease, so that it is primarily applied in cases that were not sufficiently covered by standard diagnostic approaches, i.e., ultrasound and MRI. However, involvement of extracranial vessels is present in up to 67% of cases [19,20]. For instance, Gribbons et al. reported that GCA is characterized by a diffuse pattern of inflammation in the aorta and its branches [9], with LV GCA patients having a higher recurrence rate and an increased need for an intensified therapeutic regimen [21]. In the present study, PET helped establish a diagnosis of GCA in 19/34 (55.9%) patients, most likely due to the fact that this imaging technique provides a non-invasive whole-body read-out providing information not only in carotid arteries but of the entire vasculature. In addition, in 5/7 (71.4%) of the cases in which PMR was suspected, GCA was also detected. This is of importance because GCA may be associated with significant vascular damage during follow-up [22,23,24], rendering [^18^F]FDG PET/CT as an important tool to identify such high-risk individuals initially presenting with PMR-like symptoms. Again, multiple positive predictors in the setting of PMR for identifying additional GCA using [^18^F]FDG PET/CT were identified, including low back pain, pelvic girdle and diffuse lower limb pain [25]. Such symptoms, however, may also be rather unspecific [26], further supporting the notion that [^18^F]FDG PET/CT is also useful for detecting concomitant GCA in PMR. Moreover, [^18^F]FDG PET/CT could also establish the diagnosis of PMR when the initial symptoms could not be confidently attributed. Nonetheless, the diagnosis of PMR is primarily made clinically [7], but atypical presentations are not uncommon, for example, with pain in the lumbar region or pelvic girdle that may mimic other entities such as a herniated disc or spinal claudication. In this situation, diagnosis is difficult to establish, and [^18^F]FDG PET/CT improves diagnostic accuracy [27].

The interpretation of [^18^F]FDG PET/CT in GCA and PMR remained challenging and was primarily based on a visual assessment [8,28]. However, such an approach is prone to observer bias, and a standardized definition of vascular inflammation in the setting of PMR is still lacking [8]. We, therefore, performed a semi-quantitative assessment by introducing JLR with the liver serving as a reference in a manner similar to VLR for a vascular read-out [29,30,31,32]. In our small retrospective cohort testing this novel parameter, we were able to differentiate between GCA and PMR. Furthermore, we demonstrated that VLR and JLR were significantly different from oncological controls (having no GCA and/or PMR and no signs of inflammation). Nonetheless, before JLR could be used to ascertain that an individual has PMR, future studies using JLR in a prospective setting enrolling a larger number of subjects are definitely warranted. For VLR, however, no significance was reached for differentiating between GCA vs. PMR or GCA + PMR, rendering a semi-quantitative analysis in the vessels with the liver serving as a reference as less suitable for distinguishing between subtypes of rheumatic disease [8]. As a possible explanation, (supra)aortic vessels have a relatively small diameter, so that partial volume effects may have a significant impact on the derived values, while for the joints, a larger VOI was placed [33]. However, a recent study in LVV also reported on high reliability and improved response monitoring in cases of severe inflammation when the liver was used as a reference [34]. Therefore, one may speculate whether significance for the semi-quantitative assessment for GCA may have also been reached if a larger number of subjects or different background tissue, such as the blood pool, were utilized [29]. However, with the jugular vein serving as a reference, similar results were obtained (Supplementary Materials Figure S1), further demonstrating that blood pool and liver may be suitable for calculation of target-to-background ratios in the context of GCA/PMR, which is in line with recommendations of current guidelines [8]. Nonetheless, a recent meta-analysis demonstrated that [^18^F]FDG had only moderate accuracy in detecting active sites of disease in LVV, thereby indicating that PET-based findings should be carefully interpreted in the context of clinical and laboratory parameters [35]. As such, the results of the present proof-of-concept study and herein provided ratios must definitely be confirmed in larger prospective, multi-centric settings, preferably by using phantom studies to provide harmonized semi-quantitative values among multiple PET centers [36]. Moreover, future studies may also use more leukocyte-specific radiotracers [14], such as the C-X-C motif chemokine receptor 4 targeting PET agent [^68^Ga]Pentixafor, which was found to be useful in the context of myocardial inflammation [37,38,39,40].

Our study has several limitations, including its retrospective design and a small number of investigated subjects. In addition, 4/34 [11.8%] were pre-treated with low-dose prednisolone. Current guidelines, however, endorse immediate treatment upon suspicion of GCA, and therefore, the present study may reflect a real-world clinical scenario [18,41]. Nonetheless, a bias due to prednisolone pretreatment cannot be ruled out, but diagnostic accuracy was significantly hampered in subjects receiving high-dose treatment of up to 60 mg daily (relative to the present study in with no more than 7.5 mg per day in those four individuals) [42]. In addition, further studies may also confirm PET-based findings during longer follow-up. This, however, may be challenging as not all putative sites of vessel wall inflammation can be biopsied. Moreover, more detailed information on the patient’s characteristics would be of interest, e.g., duration of symptoms. Aiming to provide a non-invasive whole-body read-out, vascular and joint targets were also chosen according to current guidelines and previously published studies [8,14], but their clinical significance has not been defined yet, and thus, future studies may also address other sites of disease. In this regard, a head-to-head comparison, e.g., with ultrasound or MRI, should also further confirm the herein presented PET-derived joint and vessel ratios. Another limitation is that this investigation focuses on patients with GCA/PMR and not on rheumatic inflammatory diseases in general. Nevertheless, GCA/PMR is most common in IUO [10], and therefore, the present study may add to the literature as we demonstrate the usefulness of [^18^F]FDG PET/CT in the context of LV GCA, in particular without typical symptoms such as vision loss or claudication of the jaw [43].

## 5. Conclusions

[^18^F]FDG-PET/CT changed the final diagnosis in the majority of patients with suspected rheumatic disease in an already highly preselected collective and was particularly useful in difficult scenarios without disease-specific manifestations. In addition, a semi-quantitative whole-body read-out of the entire vasculature and joints may also increase diagnostic certainty for the interpreting nuclear medicine physician.

## Figures and Tables

**Table 1:** Patients’ characteristics at time of scan.

Variables	Total
Clinical Parameters	
Female	20/34 (58.8)
Age (Mean ± SD)	68 ± 9.7
Symptoms	
Vision loss	1 (2.9)
Jaw claudication	3 (8.8)
Headache	4 (11.8)
Abdominal pain	6 (17.6)
Fever	10 (29.4)
Night sweats	11 (32.3)
Weight loss	12 (35.3)
Arthritis/arthralgia	13 (38.2)
-Shoulder/pelvic girdle Pain	7 (20.6)
-Pain in the spine, thigh or other presentations	6 (17.6)
Weakness	15 (44.1)
Laboratory markers of inflammation	
CRP, available in	34/34 (100)
Mean ± SD (mg/dl)	5.2 ± 5.9
abnormal (≥0.5 mg/dl)	32/34 (94.1)
ESR, available in	28/34 (82.3)
Mean ± SD (mm/1st hour)	56.2 ± 33.4
abnormal (≥50 mm/1st hour) *	16/28 (57.1)
Medication	
low dose GC (≤7.5 mg/d)	4 (11.8)
no GC	30 (88.2)

Percentages are given in parentheses. SD = standard deviation. CRP = C-reactive protein. ESR = Erythrocyte sedimentation rate. GC = glucocorticoids. * according to [12].

**Table 2:** Semiquantitative parameters.

	GCA			PMR			GCA + PMR		
	SUV_mean_	VLR	*p*	SUV_mean_	VLR	*p*	SUV_mean_	VLR	*p*
Reference Organ									
Liver (Average)	2.52 ± 0.45			2.57 ± 0.74			2.31 ± 0.42		
Arterial Segments									
Right carotid artery	2.46 ± 1.14	0.99 ± 0.46		2.49 ± 1.34	0.97 ± 0.52		2.40 ± 0.93	1.04 ± 0.40	
Left carotid artery	2.43 ± 0.80	0.46 ± 0.32		2.09 ± 0.86	0.81 ± 0.34		2.15 ± 0.84	0.91 ± 0.35	
Innominate artery	2.25 ± 0.85	0.90 ± 0.34		2.39 ± 1.41	0.93 ± 0.55		2.35 ± 0.76	0.99 ± 0.32	
Right subclavian artery	2.67 ± 1.79	1.07 ± 0.72		2.35 ± 1.72	0.92 ± 0.67		2.08 ± 1.23	0.88 ± 0.52	
Left subclavian artery	2.68 ± 1.57	1.08 ± 0.63		2.21 ± 2.05	0.86 ± 0.80		2.52 ± 1.11	1.06 ± 0.47	
Ascending aorta	2.37 ± 0.55	0.95 ± 0.22		2.42 ± 0.82	0.94 ± 0.32		3.22 ± 2.21	1.36 ± 0.93	
Aortic arch	2.36 ± 0.52	0.95 ± 0.21		2.47 ± 0.89	0.96 ± 0.35		2.13 ± 0.51	0.90 ± 0.21	
Descending aorta	2.63 ± 0.74	1.06 ± 0.30		2.63 ± 0.73	1.02 ± 0.28		2.39 ± 0.76	1.01 ± 0.32	
Abdominal aorta	2.98 ± 0.74	1.20 ± 0.30		2.71 ± 1.06	1.05 ± 0.41		2.57 ± 0.58	1.08 ± 0.24	
Right iliac artery	2.45 ± 1.25	0.98 ± 0.50		1.76 ± 0.77	0.69 ± 0.30		2.32 ± 1.04	0.98 ± 0.44	
Left iliac artery	2.53 ± 0.88	1.02 ± 0.35		2.49 ± 1.75	0.97 ± 0.68		2.23 ± 0.68	0.94 ± 0.29	
Average	2.52 ± 0.20	1.01 ± 0.08	0.07 *	2.36 ± 0.26	0.92 ± 0.1	0.09 ^†^	2.39 ± 0.31	1.04 ± 0.14	1 ^‡^
	**SUV_mean_**	**JLR**		**SUV_mean_**	**JLR**		**SUV_mean_**	**JLR**	
Joints									
Right shoulder	1.61 ± 0.53	0.64 ± 0.21		2.48 ± 1.44	1.07 ± 0.56		1.71 ± 1.00	0.74 ± 0.43	
Left shoulder	1.70 ± 0.95	0.64 ± 0.57		2.12 ± 0.92	0.92 ± 0.36		1.64 ± 0.85	0.71 ± 0.37	
SC joint (right and left)	1.39 ± 0.47	0.55 ± 0.19		1.51 ± 0.49	0.65 ± 0.19		1.64 ± 0.69	0.71 ± 0.30	
Right AC joint	1.47 ± 0.68	0.51 ± 0.14		2.35 ± 1.61	1.02 ± 0.63		1.21 ± 0.52	0.52 ± 0.22	
Left AC joint	1.29 ± 0.35	0.58 ± 0.27		1.74 ± 0.83	0.75 ± 0.32		1.06 ± 0.27	0.46 ± 0.12	
Interspinal ligaments	1.52 ± 0.54	0.60 ± 0.21		2.06 ± 0.57	0.89 ± 0.21		1.58 ± 0.78	0.68 ± 0.34	
Right ischial tuberosity	1.34 ± 0.38	0.57 ± 0.24		2.17 ± 1.06	0.94 ± 0.41		1.45 ± 0.63	0.63 ± 0.27	
Left ischial tuberosity	1.44 ± 0.61	0.53 ± 0.15		2.40 ± 1.23	1.04 ± 0.48		1.38 ± 0.80	0.60 ± 0.35	
Right greater trochanter	1.53 ± 0.45	0.59 ± 0.18		2.82 ± 1.52	1.22 ± 0.59		1.74 ± 0.73	0.75 ± 0.32	
Left greater trochanter	1.48 ± 0.46	0.61 ± 0.18		2.15 ± 1.26	0.93 ± 0.49		1.44 ± 0.47	0.63 ± 0.20	
Average	1.48 ± 0.12	0.58 ± 0.04	<0.0001 *	2.18 ± 0.37	0.94 ± 0.16	<0.0001 ^†^	1.48 ± 0.22	0.64 ± 0.09	0.08 ^‡^

* *p*-value tested for GCA vs. PMR; ^†^ *p*-value tested for PMR vs. GCA + PMR; ^‡^ *p*-value tested for GCA vs. GCA + PMR; Standardized uptake values (SUV_mean_) of liver (serving as reference) and each investigated arterial segment and joint. GCA = giant cell arteritis. PMR = polymyalgia rheumatica. VLR = Vessel wall-to-liver ratio. SC = sternoclavicular. AC = acromioclavicular.

## Data Availability

Data are not available in accordance with the European regulations regarding data protection and, therefore, cannot be provided online or via airmail. However, data are available for on-site revision.

## Supplementary Materials

The following are available online at https://www.mdpi.com/article/10.3390/diagnostics11112073/s1, Figure S1: (**a**) The Vessel to Blood Pool ratio showed no significant differences between patients with final diagnosis of giant cell arteritis (GCA) + polymyalgia rheumatica (PMR) vs. PMR or GCA alone. (**b**) The Joint to Blood Pool ratio, how-ever, was significantly increased in patients with PMR relative to GCA alone or GCA + PMR. Blood pool uptake was assessed by placing volumes of interests on jugular vein. As such, similar results were obtained when healthy liver was used as reference tissue (Figure 3). * *p* < 0.001; ns = not significant.

## References

[1] Gonzalez-Gay M.A., Vazquez-Rodriguez T.R., Lopez-Diaz M.J., Miranda-Filloy J.A., Gonzalez-Juanatey C., Martin J., Llorca J. (2009). Epidemiology of giant cell arteritis and polymyalgia rheumatica. Arthritis Care Res..

[2] Sharma A., Mohammad A.J., Turesson C. (2020). Incidence and prevalence of giant cell arteritis and polymyalgia rheumatica: A systematic literature review. Semin. Arthritis Rheum..

[3] Salvarani C., Pipitone N., Versari A., Hunder G.G. (2012). Clinical features of polymyalgia rheumatica and giant cell arteritis. Nat. Rev. Rheumatol..

[4] Franzén P., Sutinen S., von Knorring J. (1992). Giant cell arteritis and polymyalgia rheumatica in a region of Finland: An epidemiologic, clinical and pathologic study, 1984–1988. J. Rheumatol..

[5] Kermani T.A., Warrington K.J. (2013). Polymyalgia rheumatica. Lancet.

[6] Nesher G. (2014). Polymyalgia rheumatica—Diagnosis and classification. J. Autoimmun..

[7] Dasgupta B., Cimmino M.A., Kremers H.M., Schmidt W.A., Schirmer M., Salvarani C., Bachta A., Dejaco C., Duftner C., Jensen H.S. (2012). 2012 Provisional classification criteria for polymyalgia rheumatica: A European League Against Rheumatism/American College of Rheumatology collaborative initiative. Arthritis Rheum..

[8] Slart R. (2018). FDG-PET/CT(A) imaging in large vessel vasculitis and polymyalgia rheumatica: Joint procedural recommendation of the EANM, SNMMI, and the PET Interest Group (PIG), and endorsed by the ASNC. Eur. J. Nucl. Med. Mol. Imaging.

[9] Gribbons K.B., Ponte C., Carette S., Craven A., Cuthbertson D., Hoffman G.S., Khalidi N.A., Koening C.L., Langford C.A., Maksimowicz-McKinnon K. (2020). Patterns of Arterial Disease in Takayasu Arteritis and Giant Cell Arteritis. Arthritis Care Res..

[10] Schönau V., Vogel K., Englbrecht M., Wacker J., Schmidt D., Manger B., Kuwert T., Schett G. (2018). The value of ^18^F-FDG-PET/CT in identifying the cause of fever of unknown origin (FUO) and inflammation of unknown origin (IUO): Data from a prospective study. Ann. Rheum. Dis..

[11] Grayson P.C., Alehashemi S., Bagheri A.A., Civelek A.C., Cupps T.R., Kaplan M.J., Malayeri A.A., Merkel P.A., Novakovich E., Bluemke D.A. (2018). (18) F-Fluorodeoxyglucose-Positron Emission Tomography As an Imaging Biomarker in a Prospective, Longitudinal Cohort of Patients With Large Vessel Vasculitis. Arthritis Rheumatol..

[12] Hunder G.G., Bloch D.A., Michel B.A., Stevens M.B., Arend W.P., Calabrese L.H., Edworthy S.M., Fauci A.S., Leavitt R.Y., Lie J.T. (1990). The American College of Rheumatology 1990 criteria for the classification of giant cell arteritis. Arthritis Rheum..

[13] Duell J., Krummenast F., Schirbel A., Klassen P., Samnick S., Rauert-Wunderlich H., Rasche L., Buck A.K., Wester H.J., Rosenwald A. (2021). Improved primary staging of marginal zone lymphoma by addition of CXCR4-directed PET/CT. J. Nucl. Med..

[14] Sherzay R., Witte T., Derlin T., Hoepfner M., Bengel F.M. (2021). Vessel Wall Inflammatory Activity as Determined by F-18 Fluorodeoxyglucose PET in Large Vessel Vasculitis Is Attenuated by Immunomodulatory Drugs. Diagnostics.

[15] Medzhitov R. (2008). Origin and physiological roles of inflammation. Nature.

[16] Balink H., Bennink R.J., Veeger N.J., van Eck-Smit B.L., Verberne H.J. (2014). Diagnostic utility of (18)F-FDG PET/CT in inflammation of unknown origin. Clin. Nucl. Med..

[17] Ventura I., Reid P., Jan R. (2018). Approach to Patients with Suspected Rheumatic Disease. Prim. Care.

[18] Hellmich B., Agueda A., Monti S., Buttgereit F., de Boysson H., Brouwer E., Cassie R., Cid M.C., Dasgupta B., Dejaco C. (2020). 2018 Update of the EULAR recommendations for the management of large vessel vasculitis. Ann. Rheum. Dis..

[19] Prieto-González S., Arguis P., García-Martínez A., Espígol-Frigolé G., Tavera-Bahillo I., Butjosa M., Sánchez M., Hernández-Rodríguez J., Grau J.M., Cid M.C. (2012). Large vessel involvement in biopsy-proven giant cell arteritis: Prospective study in 40 newly diagnosed patients using CT angiography. Ann. Rheum. Dis..

[20] Blockmans D., Ceuninck L., Vanderschueren S., Knockaert D., Mortelmans L., Bobbaers H. (2006). Repetitive 18F-fluorodeoxyglucose positron emission tomography in giant cell arteritis: A prospective study of 35 patients. Arthritis Rheum..

[21] Muratore F., Kermani T.A., Crowson C.S., Green A.B., Salvarani C., Matteson E.L., Warrington K.J. (2015). Large-vessel giant cell arteritis: A cohort study. Rheumatology.

[22] de Boysson H., Liozon E., Lambert M., Parienti J.-J., Artigues N., Geffray L., Boutemy J., Ollivier Y., Maigné G., Ly K. (2016). 18F-fluorodeoxyglucose positron emission tomography and the risk of subsequent aortic complications in giant-cell arteritis: A multicenter cohort of 130 patients. Medicine.

[23] Espitia O., Néel A., Leux C., Connault J., Espitia-Thibault A., Ponge T., Dupas B., Barrier J.H., Hamidou M.A., Agard C. (2012). Giant Cell Arteritis with or without Aortitis at Diagnosis. A Retrospective Study of 22 Patients with Longterm Followup. J. Rheumatol..

[24] Nuenninghoff D.M., Hunder G.G., Christianson T.J.H., McClelland R.L., Matteson E.L. (2003). Incidence and predictors of large-artery complication (aortic aneurysm, aortic dissection, and/or large-artery stenosis) in patients with giant cell arteritis: A population-based study over 50 years. Arthritis Rheum..

[25] Prieto-Peña D., Martínez-Rodríguez I., Loricera J., Banzo I., Calderón-Goercke M., Calvo-Río V., González-Vela C., Corrales A., Castañeda S., Blanco R. (2019). Predictors of positive (18)F-FDG PET/CT-scan for large vessel vasculitis in patients with persistent polymyalgia rheumatica. Semin. Arthritis Rheum..

[26] De Palma M.J., Ketchum J.M., Saullo T. (2011). What is the source of chronic low back pain and does age play a role?. Pain Med..

[27] Henckaerts L., Gheysens O., Vanderschueren S., Goffin K., Blockmans D. (2018). Use of 18F-fluorodeoxyglucose positron emission tomography in the diagnosis of polymyalgia rheumatica-A prospective study of 99 patients. Rheumatology.

[28] Blockmans D., Stroobants S., Maes A., Mortelmans L. (2000). Positron emission tomography in giant cell arteritis and polymyalgia rheumatica: Evidence for inflammation of the aortic arch. Am. J. Med..

[29] Besson F.L., de Boysson H., Parienti J.J., Bouvard G., Bienvenu B., Agostini D. (2014). Towards an optimal semiquantitative approach in giant cell arteritis: An (18)F-FDG PET/CT case-control study. Eur. J. Nucl. Med. Mol. Imaging.

[30] Hautzel H., Sander O., Heinzel A., Schneider M., Müller H.-W. (2008). Assessment of Large-Vessel Involvement in Giant Cell Arteritis with ^18^F-FDG PET: Introducing an ROC-Analysis–Based Cutoff Ratio. J. Nucl. Med..

[31] Lehmann P., Buchtala S., Achajew N., Haerle P., Ehrenstein B., Lighvani H., Fleck M., Marienhagen J. (2011). 18F-FDG PET as a diagnostic procedure in large vessel vasculitis—A controlled, blinded re-examination of routine PET scans. Clin. Rheumatol..

[32] Prieto-González S., Depetris M., García-Martínez A., Espigol-Frigolé G., Tavera-Bahillo I., Corbera-Bellata M., Planas-Rigol E., Alba M., Hernández-rodríguez J., Grau J. (2014). Positron emission tomography assessment of large vessel inflammation in patients with newly diagnosed, biopsy-proven giant cell arteritis: A prospective, case control study. Ann. Rheum. Dis..

[33] Soret M., Bacharach S.L., Buvat I. (2007). Partial-Volume Effect in PET Tumor Imaging. J. Nucl. Med..

[34] Dashora H.R., Rosenblum J.S., Quinn K.A., Alessi H., Novakovich E., Saboury B., Ahlman M.A., Grayson P. (2021). Comparing Semi-quantitative and Qualitative Methods of Vascular FDG-PET Activity Measurement in Large-Vessel Vasculitis. J. Nucl. Med..

[35] Van der Geest K.S.M., Treglia G., Glaudemans A.W.J.M., Brouwer E., Sandovici M., Jamar F., Gheysens O., Slart R.H.J.A. (2021). Diagnostic value of [18F]FDG-PET/CT for treatment monitoring in large vessel vasculitis: A systematic review and meta-analysis. Eur. J. Nucl. Med. Mol. Imaging.

[36] Daisaki H., Kitajima K., Nakajo M., Watabe T., Ito K., Sakamoto F., Nakahara T., Ishibashi M., Toriihara A. (2021). Usefulness of semi-automatic harmonization strategy of standardized uptake values for multicenter PET studies. Sci. Rep..

[37] Werner R.A., Hess A., Koenig T., Diekmann J., Derlin T., Melk A., Thackeray J.T., Bauersachs J., Bengel F.M. (2021). Molecular imaging of inflammation crosstalk along the cardio-renal axis following acute myocardial infarction. Theranostics.

[38] Hess A., Derlin T., Koenig T., Diekmann J., Wittneben A., Wang Y., Wester H.J., Ross T.L., Wollert K.C., Bauersachs J. (2020). Molecular imaging-guided repair after acute myocardial infarction by targeting the chemokine receptor CXCR4. Eur. Heart J..

[39] Reiter T., Kircher M., Schirbel A., Werner Rudolf A., Kropf S., Ertl G., Buck Andreas K., Wester H.-J., Bauer Wolfgang R., Lapa C. (2018). Imaging of C-X-C Motif Chemokine Receptor CXCR4 Expression After Myocardial Infarction With [68Ga] Pentixafor-PET/CT in Correlation with Cardiac MRI. JACC Cardiovasc. Imaging.

[40] Werner R.A., Koenig T., Diekmann J., Haghikia A., Derlin T., Thackeray J.T., Napp L.C., Wester H.J., Ross T.L., Schaefer A. (2021). CXCR4-Targeted Imaging of Post-Infarct Myocardial Tissue Inflammation: Prognostic Value After Reperfused Myocardial Infarction. JACC Cardiovasc. Imaging.

[41] Dejaco C., Singh Y.P., Perel P., Hutchings A., Camellino D., Mackie S., Abril A., Bachta A., Balint P., Barraclough K. (2015). 2015 Recommendations for the management of polymyalgia rheumatica: A European League Against Rheumatism/American College of Rheumatology collaborative initiative. Ann. Rheum. Dis..

[42] Nielsen B.D., Gormsen L.C., Hansen I.T., Keller K.K., Therkildsen P., Hauge E.M. (2018). Three days of high-dose glucocorticoid treatment attenuates large-vessel 18F-FDG uptake in large-vessel giant cell arteritis but with a limited impact on diagnostic accuracy. Eur. J. Nucl. Med. Mol. Imaging.

[43] Dejaco C., Brouwer E., Mason J.C., Buttgereit F., Matteson E.L., Dasgupta B. (2017). Giant cell arteritis and polymyalgia rheumatica: Current challenges and opportunities. Nat. Rev. Rheumatol..

