# Supplementary material for: Whole-Body [18F]FDG PET/CT Can Alter Diagnosis in Patients with Suspected Rheumatic Disease
Source: Diagnostics (Basel). 2021 Nov 9;11(11):2073. doi: 10.3390/diagnostics11112073 (PMC8625716; doi:10.3390/diagnostics11112073)
Supplement: Supplementary file 1 [file diagnostics-11-02073-s001.zip › diagnostics-1412014-supplementary.pdf]

diagnostics

MDPI

Supplementary

Whole-Body [18F]FDG PET/CT Can Alter Diagnosis in Patients with Suspected Rheumatic Disease

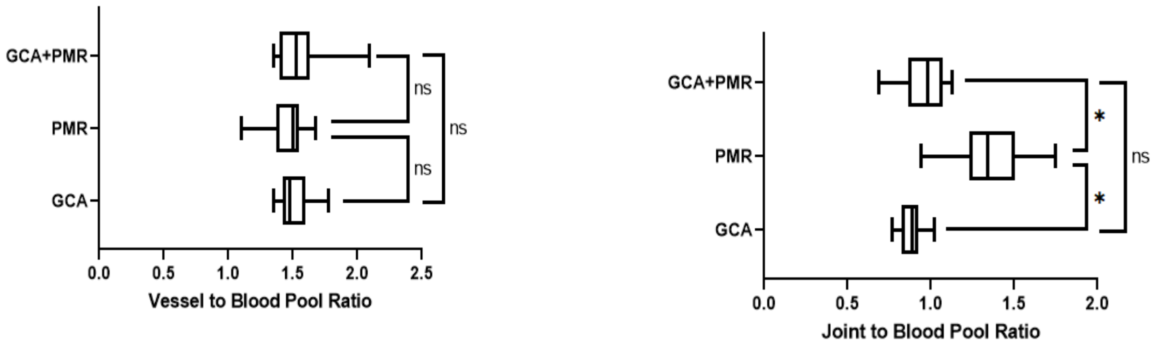

a b

Figure S1. (a) The Vessel to Blood Pool ratio showed no significant differences between patients with final diagnosis of giant cell arteritis (GCA) + polymyalgia rheumatica (PMR) vs. PMR or GCA alone. **(b)** The Joint to Blood Pool ratio, however, was significantly increased in patients with PMR relative to GCA alone or GCA + PMR. Blood pool uptake was assessed by placing volumes of interests on jugular vein. As such, similar results were obtained when healthy liver was used as reference tissue (Figure 3). *p< 0.001; ns = not significant.
